# CD206^+^ tumor-associated macrophages promote proliferation and invasion in oral squamous cell carcinoma via EGF production

**DOI:** 10.1038/s41598-019-51149-1

**Published:** 2019-10-10

**Authors:** A. S. M. Rafiul Haque, Masafumi Moriyama, Keigo Kubota, Noriko Ishiguro, Mizuki Sakamoto, Akira Chinju, Keita Mochizuki, Taiki Sakamoto, Naoki Kaneko, Ryusuke Munemura, Takashi Maehara, Akihiko Tanaka, Jun-Nosuke Hayashida, Shintaro Kawano, Tamotsu Kiyoshima, Seiji Nakamura

**Affiliations:** 1 Section of Oral and Maxillofacial Oncology, Division of Maxillofacial Diagnostic and Surgical Sciences, Faculty of Dental Science, Kyushu University, Fukuoka, Japan; 2 OBT Research Center, Faculty of Dental Science, Kyushu University, Fukuoka, Japan; 3 Department of Oral-Maxillofacial Surgery, Dentistry and Orthodontics, The University of Tokyo Hospital, Tokyo, Japan; 4 Laboratory of Oral Pathology, Division of Maxillofacial Diagnostic and Surgical Sciences, Faculty of Dental Science, Kyushu University, Fukuoka, Japan

**Keywords:** Cancer microenvironment, Oral cancer detection

## Abstract

Tumor-associated macrophages (TAMs) promote tumor progression and inhibit anti-tumor immune response by producing various mediators and preferentially express CD163, CD204, and CD206. However, the role of these TAM subsets in oral squamous cell carcinoma (OSCC) remains unclear. Here we investigated the expression and function of TAM subsets in OSCC, especially in cancer cell proliferation. Biopsy sample from 44 patients with OSCC were examined for the expression of TAM markers and EGF by immunohistochemistry. EGF production of TAM subsets isolated from OSCC patients was assessed by flow cytometry. We also examined the effect of conditioned medium from TAM subsets on the proliferation of OSCC cells. CD163^+^ cells were detected diffusely all over the tumor and connective tissue area, while CD204^+^ and CD206^+^ cells were mainly detected in/around the tumors. Flow cytometric analysis found that CD206^+^ TAMs strongly produced EGF compared with CD163^+^ and CD204^+^ TAMs. Cell proliferation and invasion of OSCC cells cultured with conditioned medium of CD206^+^ TAMs were strongly enhanced and inhibited by anti-EGFR. The number of CD206^+^ TAMs positively correlated with worse clinical prognosis. Our results revealed differences in localization and EGF production among these TAM subsets. CD206^+^ TAMs might play a critical role in the proliferation of OSCC via EGF production.

## Introduction

Oral squamous cell carcinoma (OSCC) is a tumor of the oral cavity epithelial in origin that affects more than 400,000 people per year worldwide. The mortality rate has not been improved over decades, and the 5 year survival rate is under 70%^1^. Tumor microenvironments comprise cancer cells and various normal cells including immune, inflammatory, and stromal cells^2–4^. Several studies indicated that M2 macrophages infiltrate into the tumor microenvironment and promote tumor immunosuppression, cancer progression, angiogenesis, invasion and metastasis. This phenotype of macrophage called tumor-associated macrophage^5–7^.

CD163-, CD204-, and CD206-positive macrophages are significantly associated with poor prognosis, pTNM staging and lymph node metastasis in lung cancer^8–10^, and thus CD163, CD204, and CD206 are considered as useful markers for activation of TAMs. CD163 is a hemoglobin scavenger receptor that is specifically expressed in the monocyte-macrophage system. Recently, it has been found that soluble CD163 also may be a potential diagnostic parameter for monitoring the activity of macrophage in inflammatory diseases^11^. CD204 is a Class A scavenger receptor that is involved in the pathogenesis of atherosclerosis and the pattern recognition of pathogen infection^12^. CD206 is a macrophage mannose receptor 1 that is strongly expressed in prostate adenocarcinoma, and the number of CD206-positive TAM was correlated with poor prognosis of the disease^13^.

TAMs secrete extensive amounts of proangiogenic growth factors including epidermal growth factor (EGF) are considered as the most potent source of EGF in tumor microenvironment^14,15^. EGF acts as a chemotactic factor in the tumor microenvironment and promotes the motility and invasion of tumor cells, consequently accelerating metastasis^16,17^. EGF was also shown to promote cancer cell proliferation in cultured breast cancer cells^18^.

In this study, we investigated the expressions of CD163^+^, CD204^+^, and CD206^+^ TAMs in OSCC and their association with clinical outcomes. We also examined the levels of EGF production by each TAM subset and the effects of the proliferation of OSCC cells.

## Materials and Methods

### Ethics statement

The Institutional Review Board of Center for Clinical and Translational Research of Kyushu University Hospital (IRB serial number: 27–362) authorized the study design and methods. The study was performed along with the permitted guidelines. Written informed consent was obtained from all patients and/or their legal guardians on admission and accordingly before their involvement in this study.

### Patients

Forty-four enrolled patients with primary OSCC who were treated in the Department of Oral and Maxillofacial Surgery at Kyushu University Hospital from 2005 to 2018 (mean age, 66.5 ± 10.3 years; range 35–89 years; 25 male and 19 female patients). After taking the biopsy sample, 4% buffered formalin solution was used to fix the specimens and then implanted in paraffin to make blocks. 5-μm thick sections were prepared from the paraffin-embedded specimens, hematoxylin and eosin (H&E) were used to stain the sections to confirm the diagnosis and histologic grade by a professional oral pathologist. Stage of the tumor was classified as stated in the TNM classification of the International Union Against Cancer. WHO classification was used to determine the tumor histologic grade. According to the Yamamoto-Kohama clinical classification of OSCC the pattern of tumor invasion was assessed from the H&E-stained specimens as: grade 1 = well-defined borderline; grade 2 = cords, less-marked borderline; grade 3 = groups of cells, no distinct borderline; and grade 4 = diffuse invasion (4C = cord-like type invasion; 4D = widespread type invasion). Patients type and the clinicopathological characteristics of tumor are shown in Table 1.

**Table 1:** Association of tumor-associated macrophages (TAMs) with clinicopathologic characteristics in OSCC.

	Case (%)	CD163+ cells(/HPF)	*P*-value	CD204+ cells (/HPF)	*P*-value	CD206+ cells (/HPF)	*P-*value
**Age** ^**†**^							
≤65	26 (59.1)	34.0 ± 20.4	*N.S*.	41.4 ± 20.5	*N.S*.	44.8 ± 9.9	*N.S*.
65<	18 (40.9)	32.8 ± 20.4		42.0 ± 21.0		44.4 ± 10.1	
**Gender** ^**†**^							
Male	25 (56.8)	27.8 ± 16.6	*N.S*.	41.4 ± 24.6	*N.S*.	47.0 ± 11.5	*N.S*.
Female	19 (43.2)	37.0 ± 20.4		33.4 ± 20.4		48.0 ± 7.3	
**Primary site** ^**†**^							
Tongue	24 (54.5)	25.4 ± 15.3	*N.S*.	33.0 ± 13.4	*N.S*.	47.0 ± 10.9	*N.S*.
Gingiva	10 (22.7)	36.8 ± 26.2		43.2 ± 31.5		47.5 ± 9.7	
Buccal mucosa	6 (13.6)	40.3 ± 11.2		58.4 ± 27.3		48.5 ± 5.8	
Oral floor	3 (6.8)	39.0 ± 9.2		38.0 ± 4.9		47.0 ± 12.6	
Lymph node	1 (2.3)	35.0		24.0		44.0	
**Clinical stage** ^*^							
I	7 (15.9)	23.4 ± 18.0	*N.S*.	33.0 ± 13.8	*N.S*.	47.0 ± 10.7	0.024 *r* = 0.339
II	20 (45.5)	32.0 ± 17.1		37.5 ± 19.2		47.0 ± 9.4	
III	8 (18.1)	28.3 ± 12.7		51.1 ± 29.7		48.0 ± 11.3	
IV	9 (20.4)	38.0 ± 22.2		39.0 ± 28.0		50.0 ± 7.3	
**T classification** ^*^							
T1	8 (18.1)	22.6 ± 18.9	*N.S*.	33.0 ± 17.4	*N.S*.	46.0 ± 10.0	0.047 *r* = 0.301
T2	23 (52.3)	33.0 ± 15.4		37.0 ± 16.7		47.0 ± 9.0	
T3	7 (15.9)	24.6 ± 13.2		46.2 ± 28.0		47.0 ± 11.7	
T4	6 (13.6)	43.1 ± 26.1		68.2 ± 26.6		50.1 ± 8.4	
**Cervical nodal metastasis** ^**†**^							
+	7 (15.9)	37.0 ± 22.9	*N.S*.	38.0 ± 29.1	*N.S*.	50.0 ± 7.0	0.029
−	37 (84.1)	31.0 ± 17.0		39.0 ± 22.5		47.0 ± 9.8	
**Local recurrence** ^**†**^							
+	11 (25.0)	36.0 ± 16.3	*N.S*.	41.4 ± 29.9	*N.S*.	47.0 ± 12.1	*N.S*.
−	33 (75.0)	32.0 ± 18.9		38.0 ± 20.7		48.0 ± 8.9	
**Distant metastasis** ^**†**^							
+	4 (9.0)	45.3 ± 40.3	*N.S*.	69.8 ± 40.0	*N.S*.	52.5 ± 4.2	*N.S*.
−	40 (90.9)	32.5 ± 14.7		38.0 ± 20.0		47.0 ± 10.0	
**Histological grade** ^**†**^							
Grade 1	28 (63.6)	24.4 ± 15.6	*N.S*.	35.7 ± 25.2	*N.S*.	47.0 ± 10.2	*N.S*.
Grade 2	15 (34.1)	42.0 ± 17.5		41.4 ± 20.7		48.0 ± 9.7	
Grade 3	1 (2.3)	69.0		39.0		50.0	
**Mode of invasion (YK criteria)** ^*^							
Grade 1	6 (13.6)	20.5 ± 15.2	*N.S*.	31.0 ± 14.6	*N.S*.	47.5 ± 12.9	*N.S*.
Grade 2	10 (22.7)	34.5 ± 16.5		30.8 ± 8.1		47.5 ± 9.3	
Grade 3	13 (29.5)	42.6 ± 22.5		48.0 ± 26.9		49.0 ± 10.0	
Grade 4C	9 (20.4)	27.8 ± 9.0		43.0 ± 27.0		41.0 ± 8.4	
Grade 4D	6 (13.6)	35.8 ± 14.7		45.7 ± 25.7		34.5 ± 11.4	

^*^Spearman’s rank correlation coefficient, ^†^Mann-Whitney *U*-test and Wilcoxon signed-rank test. *N.S*., not significant.

### Immunohistochemical analysis

First, we deparaffinized the sections in xylene and then hydrated by graded series of ethanol, the detail of the staining procedure were mentioned in our previous study^19^. Following primary antibodies were used to incubate the sections for overnight at 4 °C. Primary antibodies included rabbit anti-CD3 (Clone SP7; ab16669, Abcam, Tokyo, Japan, 1:200 dilution), rabbit anti-CD20 (Clone EP459Y; ab78237, Abcam, Tokyo, Japan, 1:100 dilution), mouse anti-CD163 (Clone 10D6; Novocastra, Newcastle, UK, 1:400 dilution), mouse anti-CD204 (Clone SRA-C6; TransGenic Inc, Kumamoto, Japan, 1:200 dilution), mouse anti-CD206 (Clone 5C11; Abnova, Taipei, Taiwan, 1:300 dilution), and rabbit anti-EGF (Clone ab9695; Abcam, Tokyo, Japan, 1:30 dilution). Antibody was washed by TBST and as a chromogen 100–400 µl DAB (Peroxidase Stain DAB Kit, nacalai tesque, Kyoto, Japan) was applied to each section. Finally, Mayer’s hemalum solution (Merck KGaA, Darmstadt, Germany, 1:4 dilution) was used for counterstain and then sections were washed two times for 5 min each in dH_2_O. After dehydration, sections were mounted with coverslips. The numbers of CD3-, CD20-, CD163-, CD204-, CD206-, and EGF-positive cells were counted in 4 mm^2^ sections from five independent high-power microscopic fields (400×, 0.0625 μm^2^).

### Double and triple immunofluorescence analysis

The slides were microwaved in AR6 buffer (Opal-4 Color Manual IHC kit; PerkinElmer, Waltham, MA, USA) and cooled for 30 min. Sections were then incubated in Antibody Diluent/Blocking Buffer (Opal-4 Color Manual IHC kit; PerkinElmer) for 10 min at room temperature and then incubated with primary antibodies (as listed before). Double staining was performed with CD3/EGF, CD20/EGF, CD163/EGF, CD204/EGF, and CD206/EGF, and triple staining was performed with CD163/CD204/CD206 for 2.5 h at room temperature. Samples were washed three times in TBST for 2 min each and then incubated in Polymer HRP (Ms + Rb), (Opal-4 Color Manual IHC kit; PerkinElmer) Samples were rinsed three times in TBST for 2 min each and then incubated in Opal Fluorosphore working solution (Opal-4 Color Manual IHC kit; PerkinElmer) for 10 min at room temperature. Samples were rinsed in TBST, microwaved in AR6 buffer and then mounted with VECTASHIELD MOUNTING MEDIUM FOR FLUORESCENCE WITH DAPI (Vector Laboratories, Burlingame, CA, USA). In double staining, CD3, CD20, CD163, CD204, and CD206 were labeled with Opal 520 Fluorosphore (Green) and EGF was labeled with Opal 570 Fluorosphore (Red). In triple staining CD163, CD204, and CD206 were labeled with Opal 520 Fluorosphore (Green), Opal 570 Fluorosphore (Red), Opal 690 Fluorosphore (White), respectively. For taking photomicrographs we used a light microscope with a digital camera (BZ-9000 series; Keyence, Tokyo, Japan). The ratio of EGF-positive cells in immune cells was defined as the ratio of the number of each EGF^+^ immune cells (yellow) to the whole number of each immune cells (yellow and green) in a 4-mm^2^ field of view, from five separate areas.

### Flow cytometric analysis

For detection of intracellular EGF, 5 OSCC patients peripheral blood mononuclear cells (PBMCs) (5 × 10^5^ cells/ml) were collected and cultured in PBS and then stimulated with 40 ng/ml PMA (phorbol 12-myristate 13-acetate; Wako, Tokyo, Japan), 4 μg/ml ionomycin (Ionomycin Calcium; Wako) and Brefeldin A Solution (BioLegend, San Diego, CA, USA, 1:1000 dilution) for 6 h in a moistened chamber at 37 °C with 5% CO_2_ supply.

Next, the harvested cells were washed with eBioscience Flow Cytometry Staining Buffer (Thermo Fisher Scientific, Waltham, MA, USA). After rinsing, the cells were incubated at normal temperature for 20 min in dark with PE anti-human CD163 antibodies (Clone GHI/61, IgG1, _κ_; BioLegend), FITC anti-human CD204 antibodies (Clone REA460, IgG1; Miltenyi Biotec, Bergisch, Gladbach, Germany), and PerCP/Cy5.5 anti-human CD206 antibodies (Clone 15-2, IgG1, _κ_; BioLegend). We used PE mouse IgG1, _κ_ (BioLegend), FITC REA Control (S)-VioBright antibodies recombinant human IgG1 (Miltenyi Biotec), and PerCP/Cy5.5 mouse IgG1, _κ_ (BioLegend) as isotype control antibodies. Cells were fixed and permeabilized in Fixation Buffer (BioLegend) and Intercellular Staining Perm Wash Buffer (10X) (BioLegend), followed by staining with APC anti-hHB EGF (Clone # 125923, IgG_2A_; R&D System, Minneapolis, MN, USA). APC mouse IgG_2A_ (R&D System) served as a negative control. We used BD FACSVerse™ Flow Cytometer (Franklin Lakes, New Jersey, United States) and BD FACSuite™ software for acquiring and analyzing FACS data.

### Separation and culture of TAM subsets

PBMCs from a healthy donor were collected and by using EasySep^TM^ Human Monocyte Isolation Kit (STEMCELL Technologies Inc, Vancouver, Canada) CD14^+^ monocytes were isolated. Then, the isolated CD14^+^ cells (5 × 10^6^ cells/ml) were differentiated into M2 macrophages by CellXVivo Human M2 Macrophage Differentiation Kit (R&D System) in a humidified chamber at 37 °C in a 5% CO_2_ atmosphere for 6 days. After differentiation M2 macrophages were collected and washed.

Once counting, the cells were transfer in three 15 ml tubes. Right after, the cells were incubated with FITC anti-human CD163 antibodies (BioLegend), FITC anti-human CD204 antibodies (Miltenyi Biotec), and FITC anti-human CD206 antibodies (BioLegend) at 4 °C for 10 min in dark. MACS buffer (Miltenyi Biotec) was used to rinse the sample and then incubated with Anti-FITC MicroBeads (Miltenyi Biotec) for 15 min at 4 °C. Subsequently samples were washed and sorted in three different following TAM subsets; CD163^+^, CD204^+^, and CD206^+^ cells by manual magnetic-activated cell sorting procedure with MiniMACS^TM^ Separator and Starting Kit, (Miltenyi Biotec) as stated in manufacturer’s instruction manual and earlier reports^20^. After that each TAM subsets (2.5 × 10^6^ cells/ml) were cultured in Dulbecco’s Modified Eagle Medium: Nutrient Mixture F-12 (DMEM/F12) (Life Technologies Corporation, Carlsbad, CA, USA) with 2% FBS (Sigma-Aldrich, St. Louis, MO, USA) and 1% Penicillin-Streptomycin Mixed Solution (nacalai tesque, Kyoto, Japan). Every 3 days of culture, we collected the conditioned medium (CM) from the TAM subsets and stored the CM at 4 °C.

### Enzyme-linked immunosorbent assay (ELISA)

We used CM of CD163^+^, CD204^+^, and CD206^+^ cells as samples and DMEM (Life Technologies Corporation) with 2% FBS (Sigma-Aldrich) as the control for ELISA. We concentrated the CM using the Amicon^®^ Ultra-15 3K (Merck Millipore, Burlington, MA, USA); a total of 15 ml of culture medium was centrifuged at 4000 ×g in 25 °C for 20 min to make 200 μl of sample. The samples were examined using the Human EGF Quantikine ELISA Kit (R&D Systems, Minneapolis, MN, USA) in accordance with the manufacturer’s guidelines, and EGF concentration was measured by using the Multiskan FC microplate reader (Thermo Fisher Scientific, Waltham, MA, USA) at a wavelength of 450 nm.

### Cell division assay

The OSCC cell lines HSC-2, SQUU-A, and SQUU-B were used in this study. Establishment of the HSC-2 cell line was done from metastatic cervical lymph node lesions of OSCC of floor of the mouth^21^, while SQUU-A and SQUU-B cell lines were established from recurrent tongue cancer of the same patient by orthotopic implantation^22^. Cells were stained with CFSE Cell Division Assay Kit (Cayman Chemical Company, Ann Arbor, MI, USA) as reported in the manufacturer’s instructions and then cultured in DMEM (Life Technologies Corporation) with 10% FBS (Sigma-Aldrich) for various days. After that Cell division was analyzed by using BD FACSVerse™ Flow Cytometer.

### Cell proliferation assay

HSC-2 cells (1 × 10^4^/well) were cultured in a 96-well dish with CM from CD163^+^, CD204^+^, and CD206^+^ cells for 4 days at 37 °C with 5% CO_2_ with or without Anti-EGFR antibody (Clone ab231; Abcam, Tokyo, Japan 1:100 dilution). DMEM (Life Technologies Corporation) with 2% FBS (Sigma-Aldrich) was used as a control. Cell proliferation was examined by the XTT Cell Proliferation Assay Kit (Cayman Chemical Company, Ann Arbor, MI, USA) as mentioned by the manufacturer. The color absorbance of each sample was read by using a Multiskan FC microplate reader (Thermo Fisher Scientific, Waltham, MA, USA) at a wavelength of 450 nm.

### Cell invasion assay

HSC-2 cell lines were cultured for 4 days in CM of CD163^+^, CD204^+^, and CD206^+^ cells and used for invasion assays by using Corning^®^ BioCoat™ Matrigel^®^ Invasion Chambers (Corning Incorporated, Corning, NY, USA) As follows the product’s guidelines, three Matrigel and three control inserts were prepared for a 24 well culture dish. The CM (750 μl) of CD163^+^, CD204^+^, and CD206^+^ TAM was used as a chemoattractant in each well subsequently and the insert was filled with 500 μl of DMEM (Life Technologies Corporation) without serum and HSC-2 cell line (2.5 × 10^4^ cells/insert). The cells were cultured for 22 hours in a humid chamber with 5% CO_2_ atmospheric state at 37 °C temp. Then the inserts were removed from the wells and Matrigel was cleaned off. After that cells were fixed with 100% methanol (Junsei Chemical Co.,Ltd., Tokyo, Japan) and then stained with Mayer’s hemalum solution (Merck KGaA, Darmstadt, Germany, 1:4 dilution) and Tissue-Tek eosin (Sakura Finetek Japan Co., Ltd, Tokyo, Japan) (Supplementary Method 1).

The numbers of cells on the membrane were counted in 4 mm^2^ sections from five different high-power microscopic fields (400×; 0.0625 μm^2^). The average number of cells in the Matrigel insert membrane was divided with the average number of cells in the control insert membrane, and then multiplied with 100 to calculate the invasion percentage.

### Statistical analysis

JMP software version 11 (SAS Institute, NC, USA) was used to carry out the statistical analyses. The significant differences between each group were assessed by Mann–Whitney U test, Kruskal-Wallis test, Wilcoxon test, and Spearman’s rank correlation coefficient test. For progression-free survival (PFS) and disease-specific survival (DSS) evaluation Kaplan-Meier method was used and by using the Cox hazard test curve comparisons were calculated. Receiver operating characteristic (ROC) curve was used to evaluate the sensitivity, specificity and the areas under the curve (AUC). In all analyses, P values ≤ 0.05 were considered statistically significant.

## Results

### Expression of TAM markers in OSCC tissues

**Figure 1:**
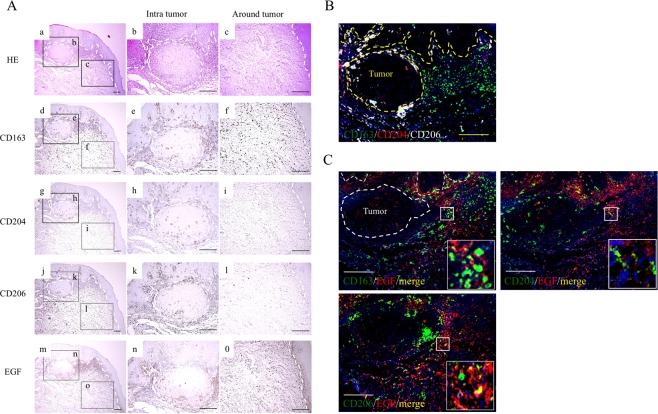
Distribution of tumor-associated macrophages (TAMs) and epithelial growth factor (EGF) in oral squamous cell carcinoma (OSCC) patients. (**A**) Representative images of paraffin sections in/around tumors stained with H&E (a–c) and CD163 (d–f), CD204 (g–i), CD206 (j–l), and EGF (m–o) antibodies (brown). Counterstaining with Mayer’s hematoxylin is shown in blue. Scale bars, 100 μm. (**B**) Triple immunofluorescence staining was performed with CD163 (green), CD204 (red), and CD206 (white); nuclei were stained with DAPI (blue). Scale bars, 50 μm. (**C**) Double immunofluorescence staining performed with EGF (red) and CD163, CD204 or CD206 (green); nuclei were stained with DAPI (blue). Merged TAM markers and EGF images (yellow). The higher magnifications are displayed at the lower right. Scale bars, 50 μm.

Immunohistochemical staining was performed to evaluate the localization of TAM markers (CD163, CD204, and CD206) in OSCC tissues. Representative histology results are shown in Fig. 1. Expression of CD163 was diffusely detected in tumor stroma and around tumors, while expressions of CD204 and CD206 were mainly detected in and around tumors (Fig. 1A). Triple immunofluorescence staining confirmed that the distribution of these TAM markers in OSCC tissues was quite different (Fig. 1B).

### Co-localization of TAM markers and the EGF proangiogenic growth factor in OSCC tissues

Immunohistochemical staining showed that the expression of EGF, a proangiogenic growth factor, was strongly detected throughout the tumors and epithelial cells (Fig. 1A). To clarify whether TAM subsets express EGF, double immunofluorescence staining with TAM markers and EGF was performed. As shown in Fig. 1C, all three TAM markers were partially co-localized with EGF. Moreover, lymphocytes (CD3 or CD20-positive cells) also were partially co-localized with EGF, but EGF was mainly expressed on TAMs, especially CD206^+^ TAMs (Supple Fig. 1).

### Expression levels of proangiogenic growth factor produced by TAMs

**Figure 2:**
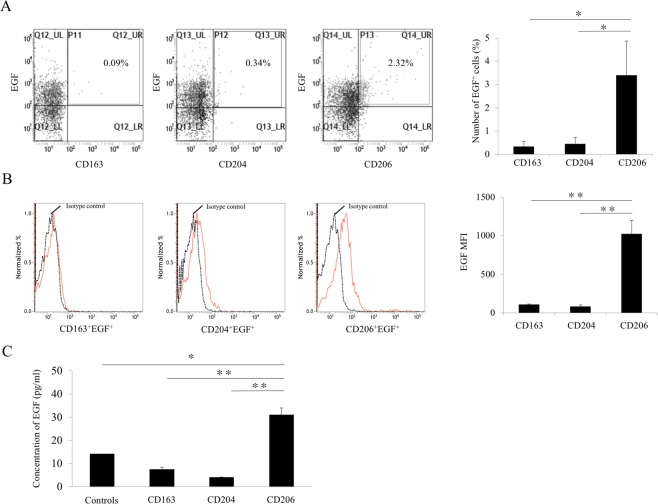
EGF expression on TAM subsets in OSSC patients. (**A**) The number of EGF^+^ cells and (**B**) EGF expression (MFI, mean fluorescent intensity) on TAM subsets. The number and MFI of EGF^+^ cells were determined using flow cytometry (n = 5 for each subset). (**C**) The concentration of EGF in conditioned medium (CM) of each TAM subset was determined by enzyme-linked immunosorbent assay (n = 7 for each subset). Cells were cultivated as described in Materials and Methods. Controls were only DMEM 2% FBS without TAM subset. Statistically significant differences between groups were determined by Kruskal-Wallis test (*P < 0.05, **P < 0.01).

To determine whether CD163, CD204, and CD206-positive cells expressed EGF, we isolated the three TAM subsets in PBMCs from 7 patients with OSCC and examined the expression levels of EGF. As shown in Fig. 2A,B, CD206^+^ cells expressed higher levels and numbers of EGF intracellularly compared with CD163^+^ and CD204^+^ cells. These results suggest a difference in the expression of EGF among the TAM subsets. To determine the production of EGF by each TAM subset, we also evaluated the concentration of EGF in the CM of each TAM subset. The concentration of EGF in CM of CD206^+^ cells was higher than that of CD163^+^ and CD204^+^ cells (Fig. 2C).

### Association of TAMs with proliferation and invasion of OSCC cell lines

**Figure 3:**
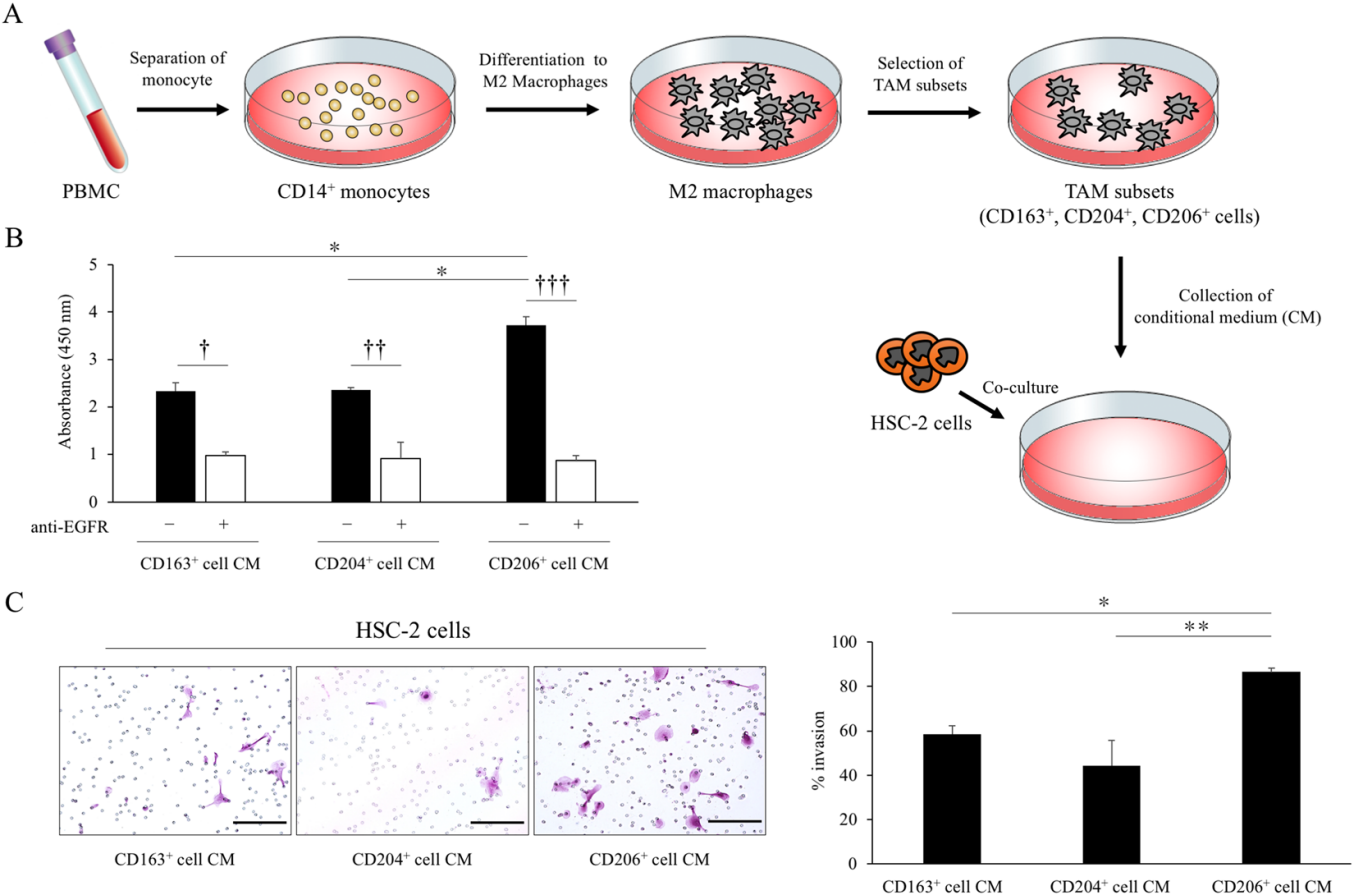
Effect of proliferation and invasion in OSCC cell lines by co-culture with CM of TAM subsets. (**A**) Scheme and representative image for the co-culture of HSC-2 cells and CM of TAM subsets for 4 days (n = 3 for each subset). Cells were cultivated as described in Materials and Methods. (**B**) Viability of HSC-2 cells co-cultured with CM of TAM subsets with/without anti-EGFR antibody (n = 3 for each subset). (**C**) Invasion activity of HSC-2 cells co-cultured with CM of TAM subsets (n = 3 for each subset). Scale bars, 100 μm. Statistically significant differences between groups were determined by Kruskal-Wallis test (*P < 0.05) and Mann-Whitney *U*-test (^†^*P* < 0.05, ^††^*P* < 0.01, ^†††^*P* < 0.001).

We next examined the effect of co-culturing CM of the TAM subsets (CD163^+^, CD204^+^, and CD206^+^ cells) with OSCC cells *in vitro* (Fig. 3A). We first evaluated cell division rates of three OSCC cell lines, including SQUU-A, SQUU-B, and HSC-2, and found that HSC-2 cells showed a high cell division rate compared with SQUU-A and SQUU-B cells (Supple Fig. 2). We therefore selected HSC-2 cells for subsequent co-culture experiments.

Cell proliferation assays revealed a highly increased viability of HSC-2 cells co-cultured with CM of CD206^+^ cells in comparison with cells co-cultured with CD163^+^ and CD204^+^ cell CM. Furthermore, we found that anti-EGFR antibody significantly reduced the viability of HSC-2 cells co-cultured with CM of TAM subsets, especially CD206^+^ cells (Fig. 3B). We also confirmed the effect of EGFR expression on HSC2 cells co-cultured with each TAM subset CM, and there were no significant differences among TAM subsets (Supple Fig. 3). Similar results were observed in invasion assays. The invasion activity of HSC-2 cells co-cultured with CM of CD206^+^ cells was more enhanced than the invasion of cells co-cultured with CD163^+^ and CD204^+^ cell CM (Fig. 3C).

### Association of TAM subsets with clinical and pathological findings of OSCC patients

We next examined the association of TAM subsets with the clinicopathologic factors of OSCC patients. Each number of TAM subsets was counted by single staining. As shown in Table 1, the number of CD163^+^ and CD204^+^ cells did not show significant associations with any clinicopathologic finding. However, the OSCC patients with clinical stage, clinical T classification, and cervical nodal metastasis showed a significant increase only in the number of CD206^+^ cells.

### Associations of TAM subsets with clinical outcomes and prognosis of OSCC patients

**Figure 4:**
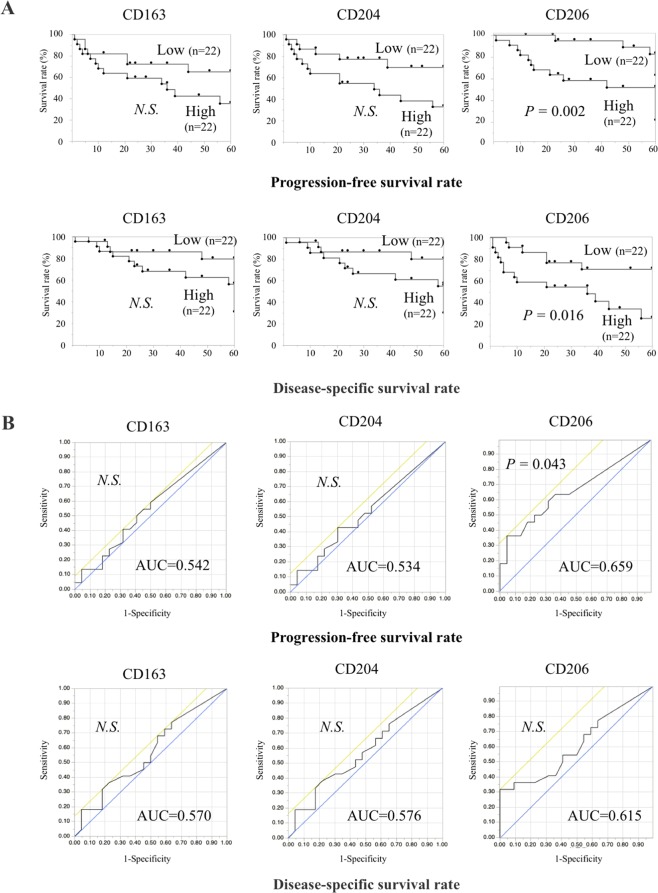
Survival curves according to the expression of TAM subsets in OSCC. Survival rates were calculated by the Kaplan-Meier method with high versus low expression of CD163^+^, CD204^+^, or CD206^+^ TAMs. The classifications are described in the Materials and Methods section. Statistically significant differences between groups were determined by log-rank test (**A**) and ROC curve (**B**).

To evaluate the correlation between TAM subsets and the clinical prognosis of OSCC patients, survival rates were estimated by using Kaplan-Meier method. We divided the OSCC patients into low and high expression groups according to the mean number of TAM subsets. In the PFS and DDS, there were no significant differences in low and high CD163^+^ and CD204^+^ expression. However, patients with high CD206^+^ expression had a significantly more unfavorable outcome than those with low expression group (Fig. 4A). According to the above results, we speculate that CD206 is a potential indicator to describe the malignancy of OSCC, so we performed statistical analysis by the ROC curve of clinical prognosis. The AUC area of DFS-related ROC curves of CD163^+^, CD204^+^, and CD206^+^ expression were 0.542, 0.534, and 0.659, respectively, and that of DSS-related ROC curves of CD163^+^, CD204^+^, and CD206^+^ expression were 0.570, 0.576, and 0.615, respectively (Fig. 4B). These results indicate higher sensitivity and specificity of CD206^+^ expression for adverse prognosis. Moreover, univariate analysis revealed that PFS and DSS were associated with YK criteria and number of CD206^+^ cells (Table 2).

**Table 2:** Univariate analysis of progression-free survival and disease-specific survival in advanced stage.

		Progression-free survival rate		Disease-specific survival rate	
		HR (95% CI)	*P* value	HR (95% CI)	*P* value
Age (y)	<65 vs 65≦	1.52 (0.62–4.06)	0.36	1.72 (0.70–4.61)	0.24
Sex	Male vs female	1.02 (0.42–2.53)	0.96	1.05 (0.42–2.54)	0.91
Pathologic tumor status	pT1 + T2 vs T3 + T4	1.45 (0.54–3.57)	0.43	2.06 (0.77–5.09)	0.15
Pathologic node status	N- vs N+	0.38 (0.06–1.35)	0.15	0.36 (0.06–1.25)	0.12
Pathologic metastasis status	M0 vs M1	2.46 (0.70–6.74)	0.14	2.48 (0.71–6.77)	0.14
Stage	1 + 2 vs 3 + 4	1.05 (0.41–2.55)	0.91	1.34 (0.52–3.24)	0.53
WHO grade	1 vs 2 + 3	1.17 (0.46–2.84)	0.72	1.40 (0.55–3.39)	0.47
YK status	1 + 2 vs 3 + 4	3.03 (1.11–10.6)	0.03^*^	3.31 (1.21–11.6)	0.02^*^
EGF positive cells	Low vs high	1.67 (0.68–4.46)	0.26	1.78 (0.72–4.73)	0.21
CD163 positive cells	Low vs high	2.21 (0.85–5.58)	0.10	2.03 (0.83–5.42)	0.12
CD204 positive cells	Low vs high	2.32 (0.95–6.18)	0.06	2.14 (0.87–5.71)	0.09
CD206 positive cells	Low vs high	3.28 (1.1–14.1)	0.03^*^	3.29 (1.1–14.1)	0.03^*^

Statistically significant differences between groups were determined by Cox proportional hazard model (*P < 0.05). HR, hazard ratio; CI, confidence interval.

Together these results suggest that CD206^+^ cells play a key role in the invasion and metastasis in OSCC.

## Discussion

In 1908, Metchnikov *et al*. first demonstrated that macrophages were efficient phagocytes and played major roles in inflammation and natural cellular immunity^23^. In the 1970’s, macrophages were considered to be notable effector cells that functioned in the cytotoxic killing of tumor cells^24^. Macrophages are divided into two functionally distinct subtypes: classically activated (M1) macrophages, which are stimulated by T helper type 1 (Th1) responses, and alternatively activated (M2) macrophages, which stimulated by Th2 responses^7,8^. M1 macrophages produce pro-inflammatory cytokines and contribute to tumor suppression, whereas M2 macrophages express anti-inflammatory cytokines and have been shown to contribute to tumors by promoting angiogenesis, immunosuppression and activation of tumor cells^25^. Recent studies have described M2-polarized macrophages as TAMs, which highly express markers, including CD163, CD204, and CD206^26–28^.

CD163 is a member of the scavenger receptor cysteine-rich family class B and is mainly expressed on mature tissue macrophages^29^. The main function of CD163 is the binding of the hemoglobin-haptoglobin complex. In addition, CD163-positive macrophages infiltrate in inflammatory tissues and were involved in the resolution of inflammation^30^.

CD204 is a prototypic member of a family of structurally different transmembrane receptors conjointly termed as scavenger receptors and is primarily expressed on macrophages and dendritic cells^31^. CD204 recognizes modified lipid proteins, and exogenous pathogen-associated molecular patterns, and apoptotic cells. We previously reported that CD163^+^CD204^+^TAMs promote T-cell apoptosis and immunosuppression via IL-10 and programmed death-ligand 1 in OSCC patients^19^.

CD206 contributes to lipid metabolism, atherogenesis, and metabolic processes^32^. CD206 is a C-type lectin, alternatively termed as the macrophage mannose receptor, that is, generally expressed by tissue macrophages, dendritic cells, and specific lymphatic or endothelial cells. CD206 plays an important role in immune homeostasis, but its high expression has been increasingly detected in the tumor microenvironment^33^.

TAMs play important functions in the tumor microenvironment, with roles in invasion, proliferation, and metastasis^34^. Several studies indicated that TAMs secrete various kinds of cytokines including growth factor, for example EGF and IL-6, and EGF strongly induces tumor cell proliferation^15–17^.

In this present study, we examined the expression of TAM subsets in OSCC tissues and the relationship between the expression of TAM markers and EGF production and tumor progression. Immunohistochemical staining demonstrated marked differences in the localizations of CD163^+^, CD204^+^, and CD206^+^ cells in OSCC tissues. Interestingly, CD206^+^ cells, from PBMCs of OSCC patients, strongly produced EGF compared with the other TAM subsets. In addition, OSCC cells showed high viability and invasion activity after co-culture with CM of CD206^+^ cells and a significant decrease in their viability with anti-EGFR antibody. These results suggest that CD206^+^ TAMs might play an important role in the proliferation and invasion of OSCC via EGF secretion. Goswami *et al*.^35^ found a paracrine loop between breast carcinoma cells and macrophages through EGF and colony stimulating factor 1 production, leading to increased carcinoma cell invasion. While this paracrine loop might be also involved in the invasion activity in OSCC cells, further examinations are needed to clarify the expression of other cytokines secreted by TAM subsets in OSCC.

Next, we examined the involvement of TAMs in the prognosis of OSCC patients. The numbers of CD206^+^ TAMs in OSCCs were positively correlated with several clinicopathologic factors including clinical stage, clinical T classification, mode of invasion, and cervical nodal metastasis. Notably, a high number of CD206^+^ TAMs was significantly correlated with poor prognosis. Recently, Dong *et al*.^36^ reported that the high presence of CD206^+^ TAMs in hepatocellular carcinoma markedly correlated with aggressive tumor phenotypes and were associated with poor prognosis. These results were consistent with our results. In contrast, in adult T-cell leukemia/lymphoma, both CD204^+^ and CD206^+^ TAMs were not associated with clinical outcome^37^. These controversial results may be due to different tumor types.

In conclusion, we demonstrated that CD206^+^ TAMs promote proliferation and invasion of OSCC cells via EGF and high numbers of CD206^+^ TAMs predict unfavorable clinical prognosis in OSCC patients. However, it is still necessary to elucidate the involvement of other cytokines secreted by TAMs in the tumorigenesis. A more detailed knowledge of the role of each TAM subset could lead to the development of novel pharmacological approaches targeting TAMs or their products, and inhibiting the tumor progression and metastasis.

## Supplementary information

Supplementary information
