# Supplementary material for: CD206+ tumor-associated macrophages promote proliferation and invasion in oral squamous cell carcinoma via EGF production
Source: Sci Rep. 2019 Oct 10;9:14611. doi: 10.1038/s41598-019-51149-1 (PMC6787225; doi:10.1038/s41598-019-51149-1)
Supplement: Supplementary file 1 — Supplementary information [file 41598_2019_51149_MOESM1_ESM.pdf]

CD206+ tumor-associated macrophages promote proliferation and invasion in oral squamous cell carcinoma via EGF production

A S M Rafiul Haque[1 *], Masafumi Moriyama[1, 2 *], Keigo Kubota[3], Noriko Ishiguro[1], Mizuki Sakamoto[1], Akira Chinju[1], Keita Mochizuki[1], Taiki Sakamoto[1], Naoki Kaneko[1], Ryusuke Munemura[1], Takashi Maehara[1], Akihiko Tanaka[1], Jun-Nosuke Hayashida[1], Shintaro Kawano[1], Tamotsu Kiyoshima[4], and Seiji Nakamura[1]

[1] Section of Oral and Maxillofacial Oncology, Division of Maxillofacial Diagnostic and Surgical Sciences, Faculty of Dental Science, Kyushu University, Fukuoka, Japan

[2] OBT Research Center, Faculty of Dental Science, Kyushu University, Fukuoka, Japan

[3] Department of Oral-Maxillofacial Surgery, Dentistry and Orthodontics, The University of Tokyo Hospital, Tokyo, Japan

[4] Laboratory of Oral Pathology, Division of Maxillofacial Diagnostic and Surgical Sciences, Faculty of Dental Science, Kyushu University, Fukuoka, Japan

* These authors contributed equally to this work.

Matrigel® invasion assay

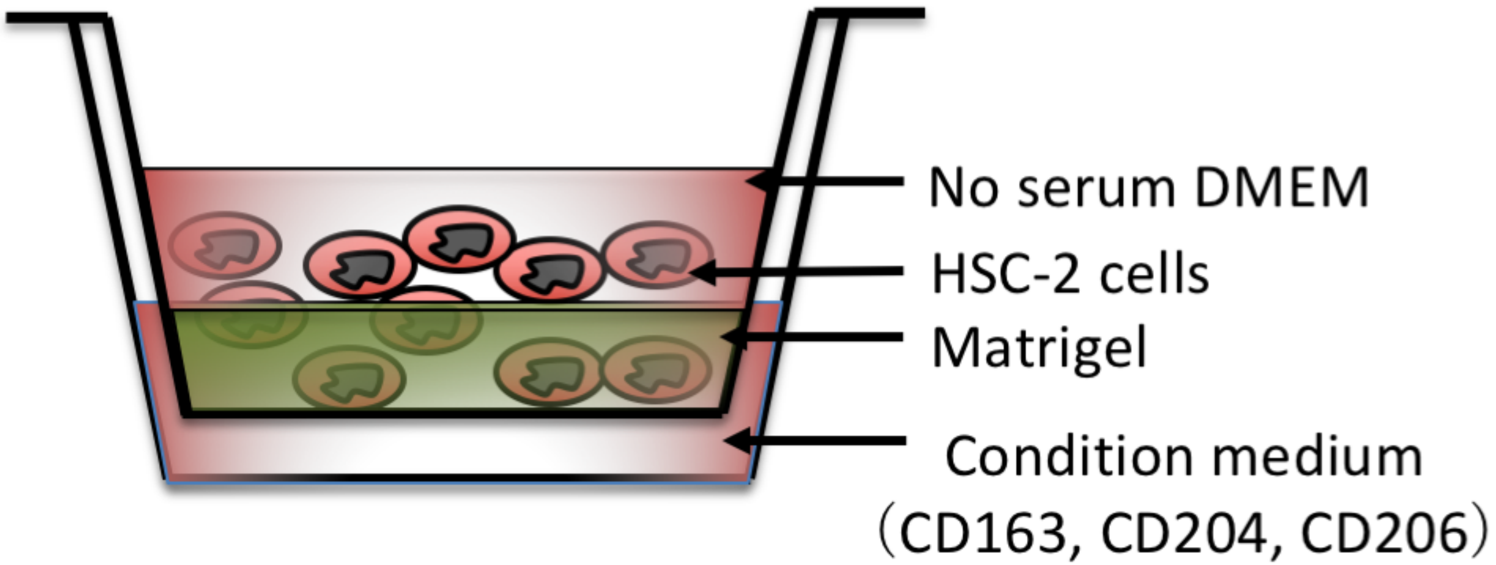

Online Supplementary Method 1

A

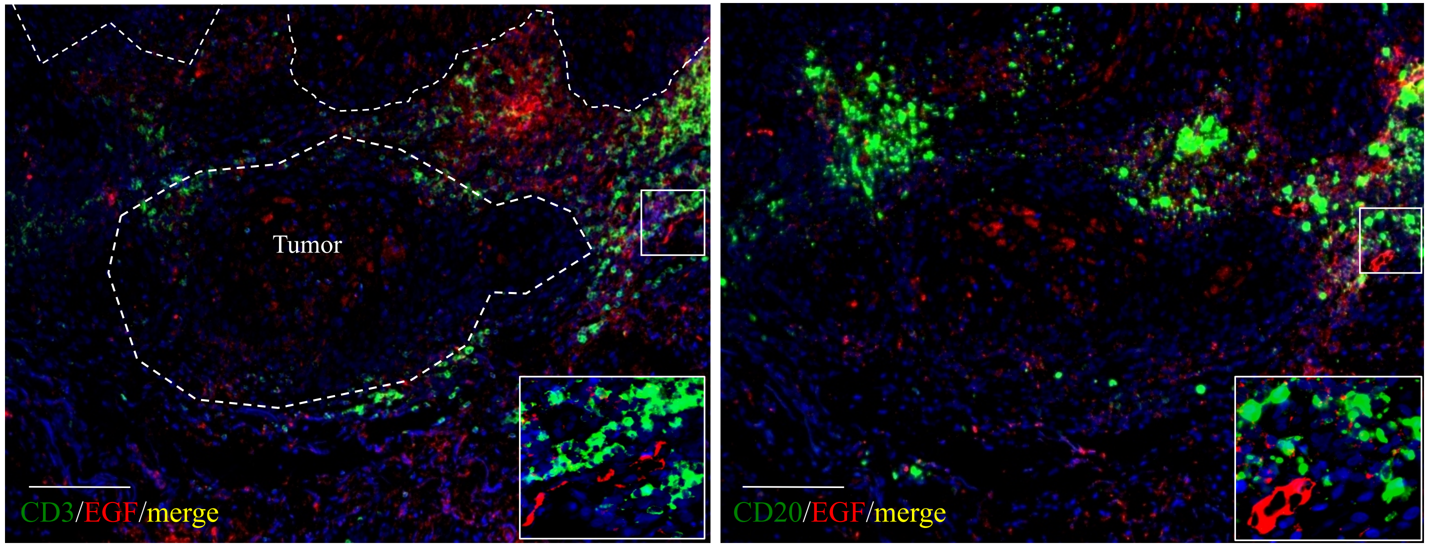

B

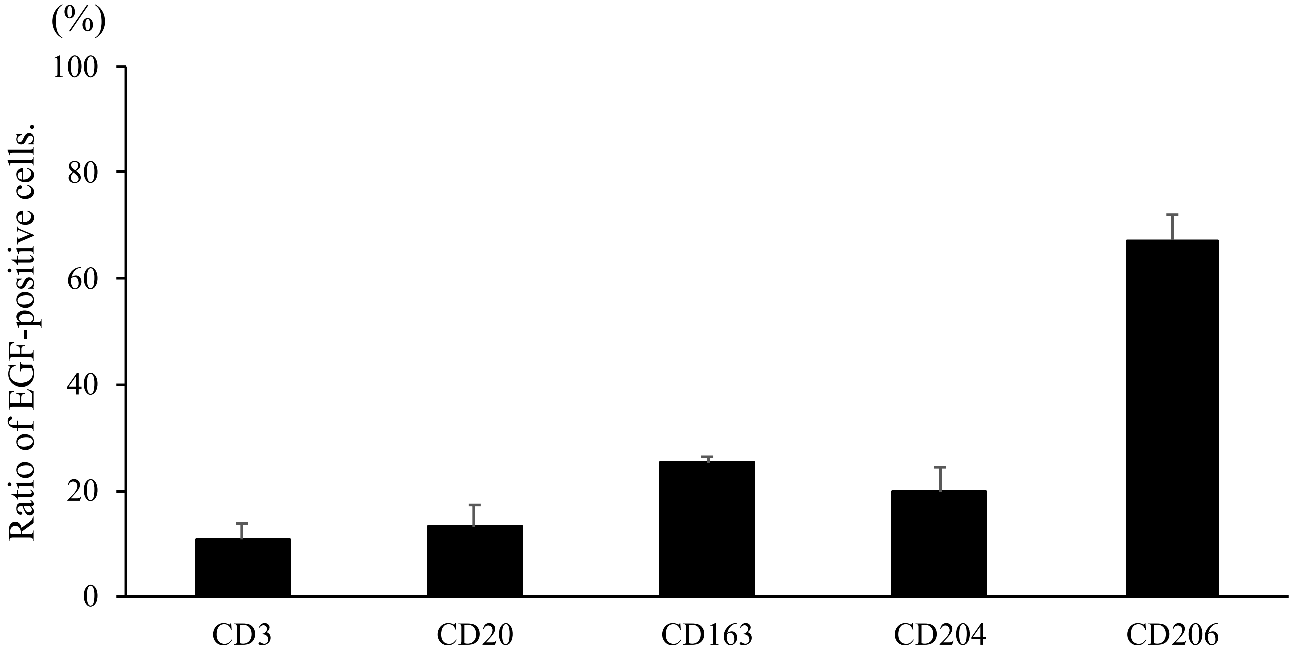

Supplementary Figure 1. Distribution of lymphocytes and EGF in OSCC patients. (A) Representative images of double immunofluorescence staining performed with EGF (red) and CD3 or CD20 (green); nuclei were stained with DAPI (blue). Merged lymphocytes and EGF images (yellow). The higher magnifications are displayed at the lower right. Scale bars, 100 µm. (B) The ratio of EGF-positive cells in immune cells from 4 patients with OSCC. The ratio was calculated as described in the Materials and methods section.

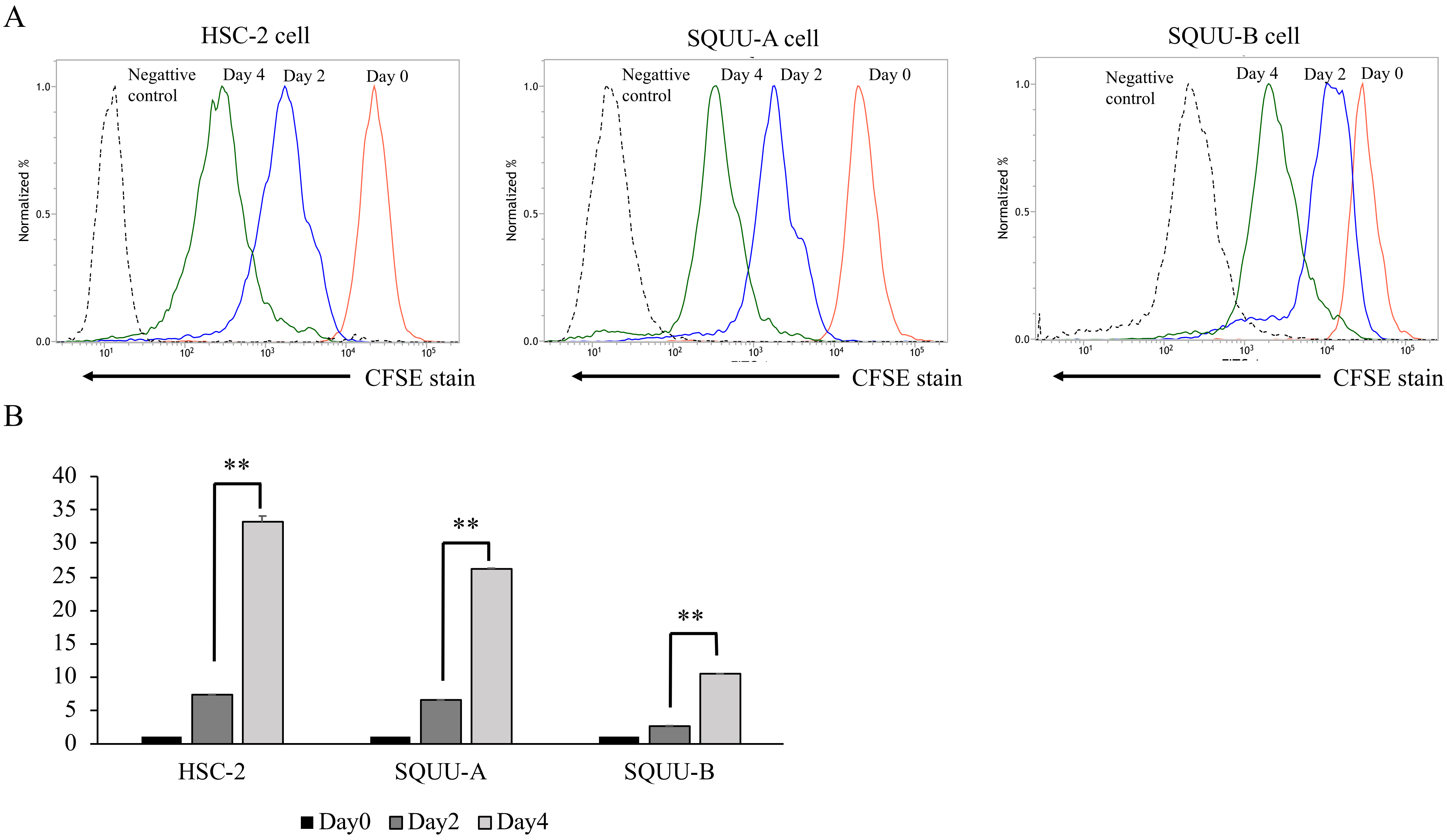

Supplementary Figure 2. Cell division of HSC-2, SQUU-A, and SQUU-B cells. Representative image for CFSE staining (A) and the division rate (B) of OSCC cell lines on day 0, 2, and 4. Cells were cultivated as described in Materials and Methods.

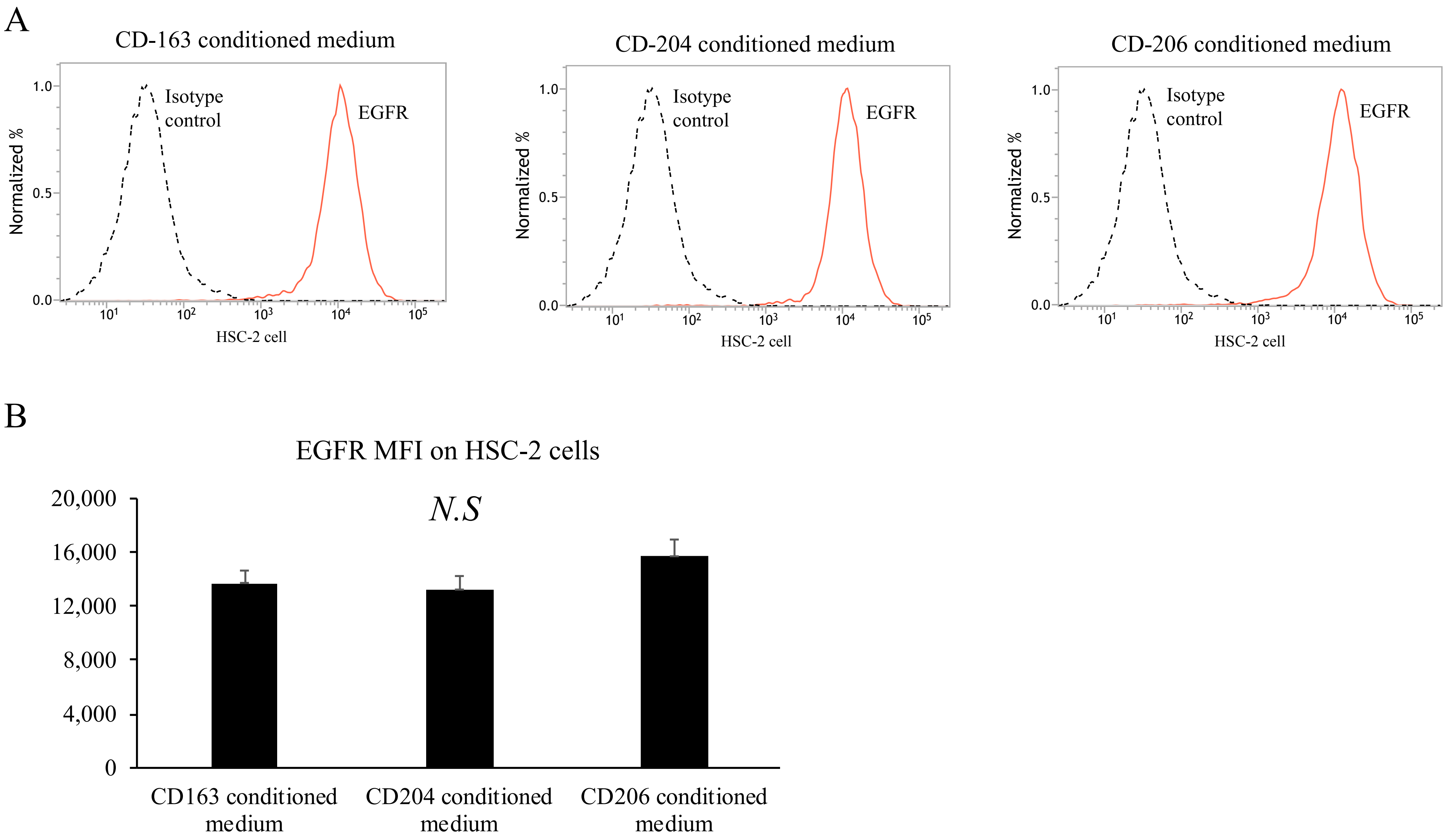

Supplementary Figure 3. Effect of EGER expression in HSC-2 cells by co-culture with CM of TAM subsets. (A) Representative image for the co-culture of HSC-2 cells and CM of TAM subsets. (B) EGFR expression (MFI) on HSC-2 cells. Cells were cultivated as described in Materials and Methods.
